# A Novel Extracellular Hsp90 Mediated Co-Receptor Function for LRP1 Regulates EphA2 Dependent Glioblastoma Cell Invasion

**DOI:** 10.1371/journal.pone.0017649

**Published:** 2011-03-08

**Authors:** Udhayakumar Gopal, Jessica E. Bohonowych, Carla Lema-Tome, Angen Liu, Elizabeth Garrett-Mayer, Bingcheng Wang, Jennifer S. Isaacs

**Affiliations:** 1 Department of Cell and Molecular Pharmacology, Medical University of South Carolina, Charleston, South Carolina, United States of America; 2 Brain Tumor Center of Excellence, Wake Forest University, Winston-Salem, North Carolina, United States of America; 3 Hollings Cancer Center Tissue Biorepository, Medical University of South Carolina, Charleston, South Carolina, United States of America; 4 Division of Biostatistics and Epidemiology, Medical University of South Carolina, Charleston, South Carolina, United States of America; 5 Rammelkamp Center for Research, Metrohealth Medical Center and Department of Pharmacology, Case Western Reserve University, Cleveland, Ohio, United States of America; The University of Chicago, United States of America

## Abstract

**Background:**

Extracellular Hsp90 protein (eHsp90) potentiates cancer cell motility and invasion through a poorly understood mechanism involving ligand mediated function with its cognate receptor LRP1. Glioblastoma multiforme (GBM) represents one of the most aggressive and lethal brain cancers. The receptor tyrosine kinase EphA2 is overexpressed in the majority of GBM specimens and is a critical mediator of GBM invasiveness through its AKT dependent activation of EphA2 at S897 (P-EphA2_S897_). We explored whether eHsp90 may confer invasive properties to GBM via regulation of EphA2 mediated signaling.

**Principal Findings:**

We find that eHsp90 signaling is essential for sustaining AKT activation, P-EphA2_S897_, lamellipodia formation, and concomitant GBM cell motility and invasion. Furthermore, eHsp90 promotes the recruitment of LRP1 to EphA2 in an AKT dependent manner. A finding supported by biochemical methodology and the dual expression of LRP1 and P-EphA2_S897_ in primary and recurrent GBM tumor specimens. Moreover, hypoxia mediated facilitation of GBM motility and invasion is dependent upon eHsp90-LRP1 signaling. Hypoxia dramatically elevated surface expression of both eHsp90 and LRP1, concomitant with eHsp90 dependent activation of src, AKT, and EphA2.

**Significance:**

We herein demonstrate a novel crosstalk mechanism involving eHsp90-LRP1 dependent regulation of EphA2 function. We highlight a dual role for eHsp90 in transducing signaling via LRP1, and in facilitating LRP1 co-receptor function for EphA2. Taken together, our results demonstrate activation of the eHsp90-LRP1 signaling axis as an obligate step in the initiation and maintenance of AKT signaling and EphA2 activation, thereby implicating this pathway as an integral component contributing to the aggressive nature of GBM.

## Introduction

High-grade astrocytoma (grade IV), or glioblastoma multiforme (GBM), is the most common and lethal of human brain cancers [1]. GBM's poor prognosis is largely attributed to the highly aggressive and infiltrative nature of these tumor cells, which invade diffusely through the brain parenchyma [2], remain following primary tumor resection [3], [4], and contribute to tumor recurrence and lethality. Therefore, alternative therapeutic modalities specifically targeting and attenuating the invasive nature of GBM are warranted.

Although numerous proteins support GBM aggressiveness, interest in the pro-motility receptor tyrosine kinase EphA2 continues to intensify. EphA2 overexpression is common in cancers, and is associated with oncogenic activity, cell invasiveness, metastatic potential and poor prognosis [5]. Clinically, EphA2 is highly overexpressed in a majority of primary and recurrent GBM specimens [6], [7], [8], and is a significant predictor of adverse outcome [7]. Although EphA2 is essential for facilitating GBM cell motility and invasion *in vitro* [9], [10], this activity is antagonized by the EphA2 ligand ephrin A1 [6], [9], [11]. Ephrin A1 suppression is frequently observed in breast and GBM specimens [6], [12], allowing these cancers to evade the restraint conferred by the inhibitory ligand. In the absence of ligand, EphA2 facilitates cell motility by coordinating signaling from a variety of RTKs via growth factor mediated activation of AKT [10], which initiates AKT-dependent EphA2 phosphorylation residue S897. This activation, resulting in P-EphA2_S897_, is required for lamellipodia formation and subsequent cell motility and invasion [10]. The inhibitory effects of ephrin A1 correlate with a disruption of EphA2-AKT complexes and loss of P-EphA2_S897_, emphasizing the importance of AKT activation and EphA2-AKT interaction for EphA2 oncogenic functions and support of GBM invasiveness.

We previously reported that the molecular chaperone heat shock protein 90 (Hsp90) supports GBM cell motility, in part by interacting with EphA2 and modulating receptor stability and function [9]. Hsp90 has a well-established intracellular role in mediating the folding and activity of numerous signaling proteins, many of which contribute to malignancy [13], [14], [15]. Hsp90 is also a reported tumor antigen [16], [17], recently explained by its extracellular localization. Mounting evidence implicates extracellular Hsp90 (eHsp90) in cancer progression given its presence in an expanding number of tumor cell types [18], [19], [20], and involvement in metastatic spread [21], [22], [23]. Although the mechanistic basis of its tumor-promoting function is not well defined, eHsp90 elicits pro-motility and pro-invasive behavior [19], [20], [21], [22], [24], [25], in concert with LRP1 [26], [27], a multi-functional receptor activated by a diverse set of ligands [28]. We now define a critical role for eHsp90 as a central regulator of EphA2-dependent GBM cell motility through its ability to sustain AKT activation and AKT-dependent activation of EphA2_S897_. Moreover, we identify a new role for LRP1 as a co-receptor for EphA2, a link strengthened by their protein interaction and co-expression in GBM specimens. Therefore, our data illuminate a novel crosstalk mechanism whereby eHsp90-LRP1 signaling is an obligate step in AKT mediated P-EphA2_S897_ activation, an event required for subsequent GBM cell invasion.

## Results

### eHsp90-LRP1 regulates EphA2 dependent motility and invasion in GBM

**Figure 1:**
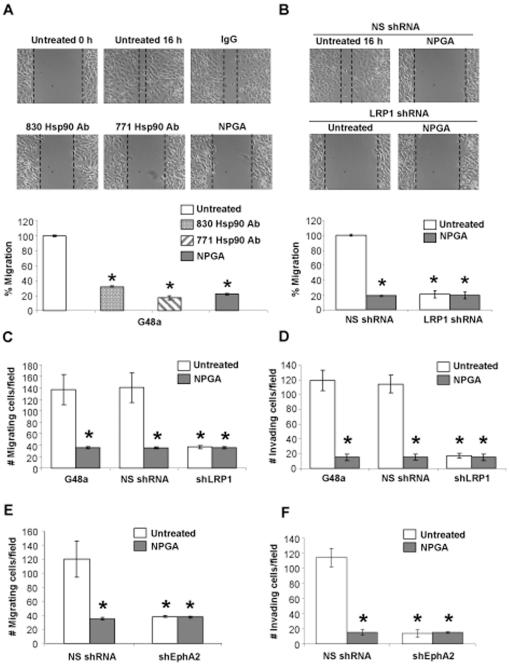
eHsp90-LRP1 regulates EphA2 dependent motility and invasion in GBM. Interference with eHsp90-LRP1 signaling inhibits GBM cell motility and invasion. (A, B) Treatment of cells with NPGA (1 µM) or anti-Hsp90 antibodies (20 ug/ml) (A), or suppression of LRP1 (B) similarly impaired G48a cell motility in wound healing assays. NS shRNA represents a nonspecifc shRNA control sequence. Percent migration is normalized to the 16 hr control and values represent the mean ± SD from 3 independent experiments (*p<0.001). (C) Serum starved parental or LRP1 silenced cells were added to top chambers of a Boyden assay and serum induced chemotaxis initiated in the presence of vehicle or NPGA. Cell numbers represent the mean ± SD from five random fields (*p<0.001). (D) The effects of eHsp90 targeting upon cell invasion were assessed by a Matrigel assay in the presence or absence of NPGA. Data is represented as the mean (± SD) of three replicates. *p<0.001. (E–F) Interference with eHsp90 function does not further inhibit cell motility or invasion in tandem with EphA2 silencing. G48a cells were transduced with either nonspecific (NS shRNA) or shEphA2 and effects of NPGA upon cell motility assessed in Boyden (right panel), and invasion assessed by Matrigel (left panel).

Despite the emerging role for eHsp90 in cancer development, nothing is known about its potential function in GBM. To investigate whether eHsp90 supports GBM aggressiveness, two approaches were utilized to block eHsp90 function. Treatment with DMAG-N-oxide, a non-permeable GA (NPGA) derivative specific for eHsp90 [21], [29] potently inhibited G48a cell motility (70%) (Figure 1A). Alternatively, antibody mediated neutralization of eHsp90 function [21], [22], [30] similarly inhibited motility. These effects were reproducible in other GBM cell lines (data not shown) and highlight a pivotal role for eHsp90 function in supporting GBM motility. eHsp90 signaling is transduced via the multifunctional LRP1 receptor [26], [27] and LRP1 has been implicated in GBM cell motility and invasion [31]. LRP1 silencing [26], (Figure S1A) blocked GBM cell motility in a manner similar to NPGA or Hsp90 antibody treatments, with no further suppression elicited by NPGA (Figure 1B). These results indicate that the pro-motility function of eHsp90 in GBM is mediated through LRP1, a conclusion further strengthened by similar trends obtained with Boyden motility and Matrigel invasion assays (Figure 1C, D, and Figure S1B, C).

To provide evidence for an eHsp90-LRP1 signaling complex in GBM, we evaluated the surface expression of Hsp90 and LRP1 in a panel of cell lines. Surface expression of Hsp90 and LRP1 was elevated in three GBM cell lines (G48a, U87, U251) in comparison to normal astrocytes (SVGA) (Figure S1D), trends consistent with their Hsp90 secretion profile (Figure S1E). Interestingly, low surface expression of Hsp90 in astrocytes correlated with its nominal LRP1 surface expression; however, its total expression level was comparable to that in GBM cells (Figure S1F), suggesting that GBM cells may preferentially translocate LRP1 to the cell surface. To explain the ability of NPGA to suppress signaling and pro-motility function, we examined whether NPGA may attenuate surface expression of eHsp90, As shown (Figure S1G), NPGA decreased surface Hsp90 levels in G48a and U87 cells (4-fold and 3-fold, respectively), with no corresponding reduction of surface LRP1 expression. The genetic silencing of LRP1 in G48a elicited a comparable decrease in Hsp90 surface expression (5-fold), strengthening the notion that surface Hsp90 levels correlate with relative surface LRP1 expression. The ability of NPGA to reduce surface expression of eHsp90 is therefore likely due to its ability to interfere with eHsp90 interaction with LRP1. It is well established GA has the capacity to dramatically alter the conformation of intracellular Hsp90 [32]. Our data therefore suggest that eHsp90 associates with LRP1 in a conformationally specific manner and that NPGA promotes an eHsp90 conformation incompatible with LRP1 binding. This notion is supported by examples where addition of agents capable of perturbing eHsp90 function prevented the ability of eHsp90 to associate with binding partners [19], [20], [23].

We, and others, have demonstrated that EphA2 plays a pivotal role in coordinating GBM cell motility and invasion [6], [9], [10]. Given our current data that eHsp90 and LRP1 are supporting partners of GBM motility, we next investigated whether the eHsp90-LRP1 pathway crosstalks with EphA2 signaling. As expected, EphA2 silencing dramatically inhibited G48a cell motility and invasion (Figures 1E, F). In support of crosstalk between the two signaling axes, NPGA did not further inhibit cell motility or invasion in EphA2 silenced cells (Figures 1E, F, Figures S2A-D). Taken together, our data implicate eHsp90-LRP1 function as a critical component of EphA2 driven motility and invasion.

### Extracellular Hsp90 signaling regulates AKT activation, subsequent AKT dependent EphA2 phosphorylation, and lamellipodia formation

**Figure 2:**
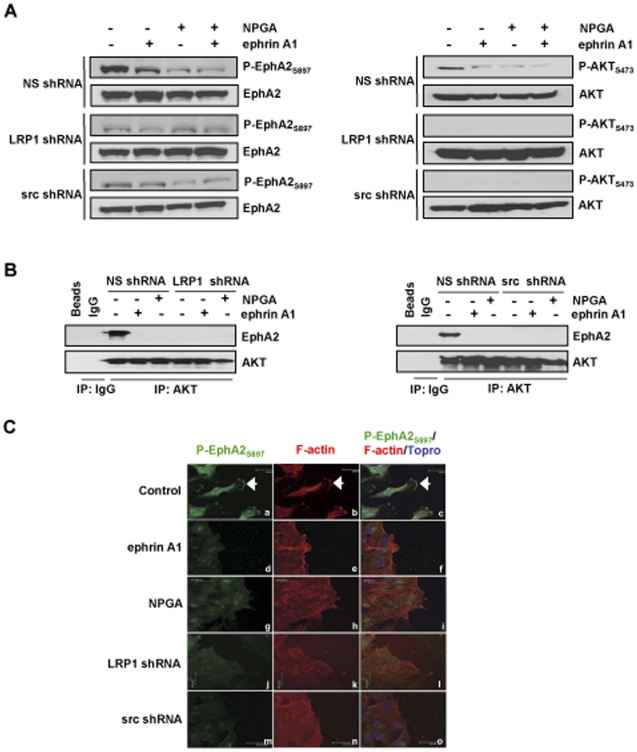
eHsp90-LRP1 signaling regulates activated EphA2 (phospho-S897), its association with AKT, and lamellipodia formation. (A) Control or LRP1 silenced G48a cells were treated with NPGA, ephrin A1, or the combination, and the effects upon P-AKT_S473_ and P-EphA2_S897_ were evaluated. The effect of src silencing was included for relative comparison. (B) Interference with eHsp90 signaling (NPGA or LRP1 silencing), or AKT activation (src silencing or treatment with ephrin A1) disrupts EphA2-AKT protein complexes. (C) G48a cells grown were fixed 4 hr after wounding, followed by immunostaining with the indicated antibodies. Arrows indicate the leading edge localization of P-EphA2_S897_ (a–c). Stimulation of cells with ephrinA1 was included as positive control for suppression of EphA2_S897_ phosphorylation (d–f). Lamellipodia formation and concomitant localization of P-EphA2_S897_ is similarly suppressed by NPGA (g–i) or LRP1 silencing (j–l), or by src silencing (m–o). Scale bars 25 µm.

To further solidify the possibility of pathway crosstalk between eHsp90 signaling and EphA2, we evaluated whether NPGA specifically impacted upon EphA2 activation. To explore this, we determined whether eHsp90 modulated the phosphorylation status of EphA2_S897_. Perturbation of eHsp90 signaling by either NPGA or LRP1 silencing effectively suppressed P-EphA2_S897_ (Figure 2A). EphA2 was recently identified as a substrate for AKT in GBM, wherein AKT-directed phosphorylation of EphA2 at S897 is required for EphA2 dependent cell motility [10]. NPGA markedly suppressed, and LRP1 silencing abrogated, AKT activation, demonstrating that perturbation of eHsp90 signaling suppresses AKT activation and concomitant AKT mediated phosphorylation of EphA2. AKT phosphorylation can be induced by src [33], and we found that NPGA or LRP1 silencing suppressed src activation (Figure S2E). To define the potential role of src in modulating P-EphA2_S897_, src was silenced (Figure S2F), which dramatically suppressed both P-AKT_S473_ and P-EphA2_S897_ (Figure 2A). This suggests that eHsp90-LRP1 mediated src signaling is a prerequisite for subsequent serine phopshorylation of AKT and EphA2.

Although treatment of cells with ephrin A1 ligand similarly inhibited AKT activation and P-EphA2_S897_, src was induced by this treatment (Figure S2G), an outcome consistent with other reports [10], [34], [35]. Although this appears inconsistent with the ligand independent role of src, the ability of ephrin A1 ligand to inhibit AKT activation is reported to be src independent and to require EphA2 kinase activity [35]. Regardless of this complexity, our results support a model whereby suppression of AKT and P-EphA2_S897_ represents a common mechanism by which eHsp90 inhibition and ephrin A1 blocks cell motility and invasion. These inhibitory effects upon EphA2, AKT, and src were recapitulated by addition of Hsp90α antibody (Figure S2G), supporting the notion that eHsp90 is a critical regulator of these pathways. We confirmed that the effect of eHsp90 perturbation upon EphA2 signaling was not due to a reduction of surface EphA2 expression (Figure S2H).

We next evaluated the consequences of the molecular changes elicited by perturbation of eHsp90 signaling. Given that NPGA or LRP1 silencing suppressed AKT-directed EphA2 phosphorylation, we examined whether eHsp90 modulated EphA2-AKT complex formation. NPGA or LRP1 silencing potently abrogated interaction between these proteins (Figure 2B). Ephrin A1 promoted a similar disruption of this complex, as expected [10], supporting the notion that regulation of this complex is a central component of GBM cell motility and invasion. As activation of P-EphA2_S897_ and its localization within lamellipodia are essential determinants for the cell motility and invasion associated with GBM [10], we next examined whether these treatments affected P-EphA2_S897_ subcellular localization. Although P-EphA2_S897_ is detected in lamellipodia in control cells following serum stimulation (Figure 2C, panel a–c), ephrin A1 abrogated P-EphA2_S897_ expression and lamellipodia formation (panels d–f), as previously reported [10]. NPGA treatment or genetic silencing of LRP1 or src, similarly abrogated receptor phosphorylation, and lamellipodia formation (panels g–o).

### ATPase deficient Hsp90 sustains AKT mediated EphA2 phosphorylation, lamellipodia formation, and cell motility in the presence of NPGA

**Figure 3:**
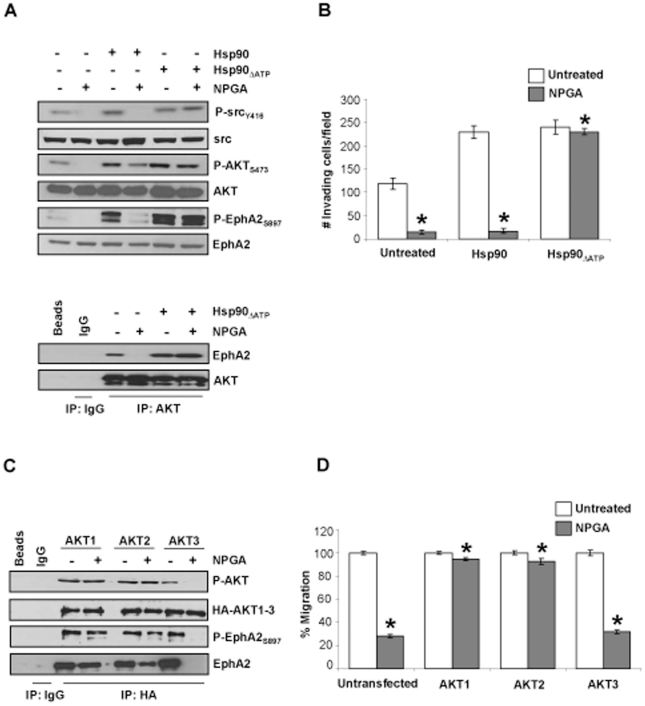
Preservation of AKT activation is required for lamellipodia formation, and concomitant cell motility and invasion. (A) Serum starved (8 hr) G48a cells were exposed to either native or Hsp90_ΔATPase_ proteins (3 µg/ml) for 15 min in the presence or absence of NPGA, and the indicated signaling molecules evaluated by immunoblot. The effect of these treatments upon EphA2-AKT interaction was also evaluated. (B) A Matrigel invasion assay was utilized to evaluate the ability of native or Hsp90_ΔATPase_ proteins to sustain cell invasion in the presence of NPGA. Values represent the mean (± SD) of 3 independent experiments. (C) G48a cells stably transduced with the indicated HA-tagged myristolyated AKT constructs were exposed to NPGA and HA immunopurified complexes were evaluated for P-AKT and P-EphA2_S897_. (D) The pro-motility function of the indicated AKT proteins was evaluated in the presence or absence of NPGA using a scratch wound assay.

To further strengthen the premise that eHsp90 signaling is essential for P-EphA2_S897_, we examined whether addition of Hsp90 protein was sufficient as a stimulus to activate this pathway. Addition of exogenous Hsp90α to serum starved G48a cells robustly stimulated phosphorylation of src, AKT and EphA2 (Figure 3A). The N-terminal ATPase domain of Hsp90 is dispensible for its extracellular pro-motility functions [26]. Our data support this notion, as addition of an N-terminally truncated Hsp90 protein (Δ1-235 aa) lacking its ATPase domain (Hsp90_ΔATP_) [26] activated P-src_Y416_, P-AKT_S473_ and P-EphA2_S897_ to an extent comparable to that of the wild type Hsp90α protein (Figure 3A). To validate the specificity of NPGA's effects upon eHsp90, we utilized NPGA in tandem with Hsp90_ΔATP_. Since the truncated protein lacks the NPGA binding pocket, this protein should not undergo a drug mediated conformational change and would therefore be expected to be resistant to the effects of drug. In support of this premise, Hsp90_ΔATP_ rescued cells from NPGA's suppressive effects upon src, AKT and EphA2. Moreover, the ability of Hsp90_ΔATP_ to preserve P-AKT_S473_ upon drug challenge further illustrates the specificity of NPGA for eHsp90, as cell permeable Hsp90 inhibitors suppress AKT activation [36]. We next examined whether the Hsp90_ΔATP_ mediated activation of EphA2 and AKT correlated with its ability to foster association between these proteins. Hsp90_ΔATP_ sustained EphA2-AKT complexes during challenge with NPGA (Figure 3A), and elicited a modest increase in their association, indicating that eHsp90 expression influences the magnitude of downstream signaling and augment the affinity of EphA2 for its binding partners.

We next performed a more thorough analysis to determine the extent to which Hsp90_ΔATP_ protected cells from NPGA's suppressive effects. Interestingly, both wild type and Hsp90_ΔATP_ protein comparably increased G48a cell invasion by two-fold. However, whereas NPGA treatment abrogated cell motility and invasion of cells fortified with native protein, Hsp90_ΔATP_ rescued cells from these inhibitory effects (Figure 3B and Figure S3A, B). We extended the relevance of these findings by evaluating P-EphA2_S897_ and lamellipodia formation in intact cells. Addition of native or Hsp90_ΔATP_ protein to starved G48a cells potently stimulated P-EphA2_S897_ and lamellipodia formation (Figure S3C panels d–f, j–l). Importantly, while NPGA suppressed P-EphA2_S897_ in the presence of native protein, this agent had no inhibitory effects upon cells treated with Hsp90_ΔATP_, evidenced by the retention of both P-EphA2_S897_ and lamellipodia formation (panels g–i and m–o). These data are congruous with our molecular data (Figure 3A), and strongly implicate activation of P-AKT_S473_ and P-EphA2_S897_ as essential components of eHsp90-dependent lamellipodia formation and cell motility and invasion.

To further test the premise that eHsp90 mediated activation of AKT was essential for its pro-motility function, we next examined whether constitutive activation of AKT would antagonize the effects of NPGA. To explore this, G48a cells were transduced with constitutively active myristolated AKT (Myr-AKT) proteins [37]. Myr-AKT1 and Myr-AKT2 maintained their activated P-AKT status when challenged with drug, as well as their interaction with P-EphA2_S897_ (Figure 3C). Interestingly, although these three isoforms share a large degree of homology [38], Myr-AKT3 did not sustain its activation status, P-EphA2_S897_ expression, or interaction between EphA2 and AKT following NPGA challenge. Importantly, the ability of AKT isoforms to maintain P-EphA2_S897_ occurs concomitantly with their ability to rescue cells from the anti-motility effects of NPGA. Myr-AKT1 and Myr-AKT2 transduced cells retained their motile properties in the presence of NPGA, while motility was suppressed in drug treated Myr-AKT3 transduced cells, concordant with suppressed Myr-AKT3 activation (Figure 3D, Figure S3D). While the basis of NPGA mediated inactivation of Myr-AKT3 is currently unknown, our results are consistent with the premise that eHsp90 mediated activation of AKT is an essential component of EphA2 directed cell motility.

### Hypoxia stimulates GBM motility and invasion via amplified eHsp90-LRP1 signaling and consequent activation of AKT and EphA2

**Figure 4:**
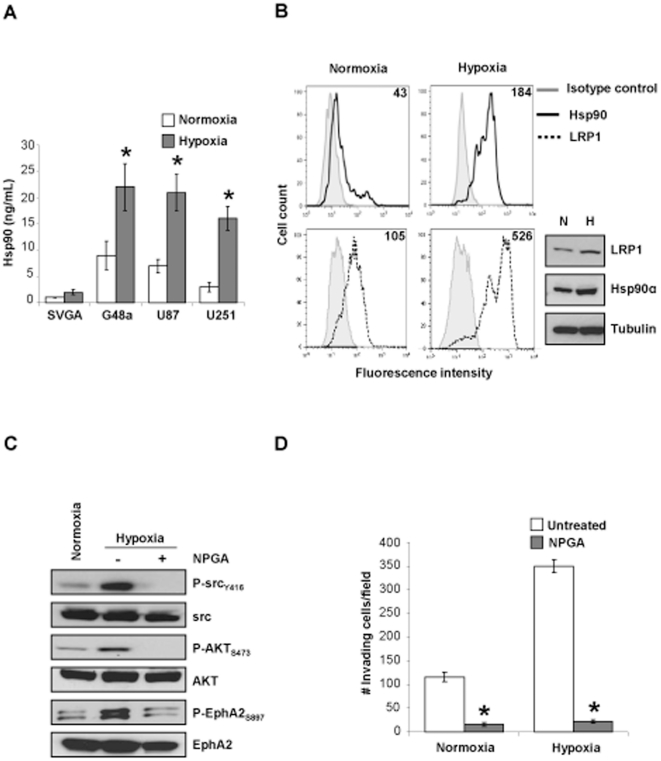
Hypoxic conditions amplify eHsp90-LRP1 initiated AKT-EphA2 signaling. (A) The ability of hypoxia to modulate Hsp90α secretion was determined by ELISA, as in Supplementary Figure S1E. (B) G48a cells cultured in 1% serum were exposed to hypoxia (1% O_2_) for 36 hr and surface expression of Hsp90α and LRP1 was determined in intact cells by flow cytometry. A corresponding immunoblot shows total cellular expression of LRP1 and Hsp90α. (C) Representative immunoblot demonstrating effects of hypoxia upon activation of src, AKT, and EphA2 in the presence or absence of NPGA. (D) A Matrigel invasion assay was utilized to evaluate the effects of hypoxia (16 hr) upon cell invasion in the presence or absence of NPGA.

Tumor hypoxia, a hallmark of GBM [39], is a well known enhancer of cell motility and invasiveness [24], [40]. As hypoxia induces both Hsp90 secretion and cell motility [24], we investigated whether hypoxia utilizes eHsp90 dependent signaling to promote GBM motility and invasion. Hypoxia elicited a 2.5-fold increase in Hsp90α secretion in G48a cells, with similar dramatic increases observed in U87 and U251 GBM cells (Figure 4A). Furthermore, hypoxia increased surface expression of both Hsp90 and LRP1 (4-fold and 5-fold, respectively) (Figure 4B). Interestingly, cellular expression of both LRP1 and Hsp90α was also elevated (2-fold, and 1.5 fold, respectively). To examine whether hypoxia-mediated increases in eHsp90 and LRP1 amplified the eHsp90-LRP1 signaling axis, we surveyed the activation status of the signaling intermediates src, AKT and EphA2. Hypoxia robustly activated src, AKT and EphA2 (11.4, 5.6, and 8.4-fold, respectively) (Figure 4C), and concomitantly induced cell motility (40%) and invasion (3.5-fold) (Figure 4D, Figure S4A, B). Strikingly, NPGA completely abrogated hypoxia stimulated activation of src, AKT, and EphA2, and suppressed cell motility and invasion (Figure 4D, Figure S4A, B). These results clearly demonstrate that hypoxia dependent upregulation of surface LRP1 and Hsp90 potentiate eHsp90-LRP1 signaling to facilitate AKT activation and subsequent EphA2 directed cell motility.

### LRP1 is a co-receptor for EphA2 and co-localizes with P-EphA2_S897_ in clinical GBM specimens

**Figure 5:**
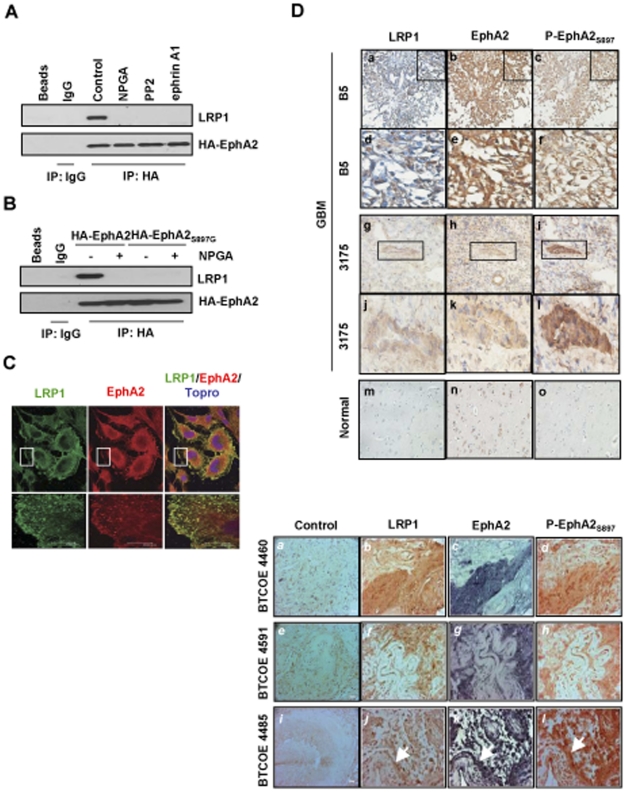
LRP1 is a co-receptor for EphA2 and co-localizes with P-EphA2_S897_ in clinical GBM specimens. (A) HA-EphA2 transfected U87 cells were treated with NPGA, ephrin A1, or the src inhibitor PP2 for 16 hr. LRP1 was detected from HA immunoprecipitates. (B) U87 cells were transfected with HA-tagged wild type or point mutant (S897G) EphA2 plasmid, and LRP1 was detected from HA immunoprecipitates. (C) U87 cells were immunostained with indicated antibodies showing that LRP1 co-localized with EphA2. Scale bar 25 µm. The bottom panels represent magnified areas of confocal images derived from the respective upper panels, as delineated by the boxed region. Scale bar 5 µm. (D) Detection of P-EphA2_S897_ and LRP1 in human GBM specimens. Panels a–f, and m–o are paraffin sections, panels g–l are frozen specimens. Magnification ×200. Lower panels (*a–l*) are paraffin sections from the recurrent GBM specimens. Magnification ×400.

Given that LRP1 is required for eHsp90 dependent EphA2 activation, we next investigated whether this regulation is mediated by a physical interaction. To explore this, U87 cells, which express high levels of LRP1 (Figure S1F), were transduced with HA-tagged EphA2 plasmids. Interestingly, we demonstrate a robust association between LRP1 and EphA2 in untreated cells. This interaction is similarly disrupted by treatments that suppress AKT activation (NPGA, PP2, or ephrin A1), (Figure 5A). Given that AKT activation facilitates P-EphA2_S897_, we next asked whether phosphorylation of S897 on EphA2 was required for its interaction with LRP1. To examine this, we utilized an EphA2 point mutant protein (S897G), which is not recognized by the P-EphA2_S897_ specific antibody (Figure S5). As shown in Figure 5B, EphA2_S897G_ was unable to interact with LRP1, reinforcing our hypothesis that the AKT consensus site of EphA2 represents a critical recognition motif required for its interaction with LRP1.

To further examine the potential co-receptor function of LRP1, we next utilized fluorescence microscopy to determine the proximity of LRP1 and EphA2 in cells. In support of our biochemical results, EphA2 and LRP1 exhibited a significant degree of co-localization (Figure 5C), especially at the leading edge of GBM cells. To explore the clinical relevance of this finding, we examined the pattern of LRP1 and EphA2 staining in serial sections derived from clinical GBM specimens, and observed a consistently overlapping pattern of LRP1 and P-EphA2_S897_ (Figure 5D, top panel, a–f). Expression of P-EphA2_S897_ has been reported within areas of vascularity in GBM specimens [10], and we similarly observe P-EphA2_S897_ in the microvasculature, along with prominent perivascular immunoreactivity for LRP1 (panels g–l). In the lower set (panels *j–l*, indicated by arrows), an overlapping staining pattern of LRP1, EphA2, and P-EphA2_S897_ is visible at the periphery of a blood vessel, as well as within the vessel lumen, indicative of cell invasion. The consistent trends of expression of these proteins within similar proximity strongly suggest a functional interaction in GBM. BTCOE specimens 3175, 4460, 4591, and 4485 were derived from recurrent GBM.

In contrast to the modest expression of P-EphA2_S897_ and LRP1 in GBM tissues, nominal expression was noted in normal brain tissue (panels m and o), and EphA2 expression was similarly weak (panel n). To evaluate the prevalence of LRP1 expression in GBM, we examined a larger cohort (75) of GBM specimens. While the majority (91%) of normal tissues was negative for LRP1, 68% of Grade IV GBM tissues exhibited moderate to high expression (Table 1). Fisher's exact test indicated a statistically significant association between sample type and LRP1 expression level among these groups (P<0.001). These data support a prior report documenting higher LRP1 expression in a variety of brain derived neoplasms [41]. Our data strengthen the notion that LRP1 contributes to the pathological nature of this disease, in large part via promotion of EphA2 dependent signaling.

**Table 1:** Distribution of LRP1 expression in normal and GBM specimens.

Expression	Normal (%)	Glioblastoma (%)	Total (%)
Negative	21 (91)	7 (9)	28 (29)
Low	2 (9)	17 (23)	19 (19)
Moderate	0 (0)	30 (40)	30 (31)
High	0 (0)	21 (28)	21 (21)
Total	23 (100)	75 (100)	98 (100)

Histopathological scoring is as follows: negative staining (0), weak staining (1), moderate (2–3), and strong (4–5). Fisher's exact test suggests a strong association between sample type and LRP1 expression level (p<0.001).

## Discussion

**Figure 6:**
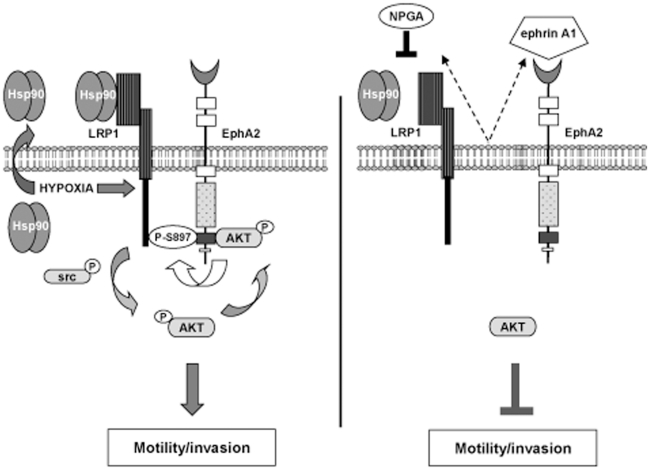
Molecular crosstalk between eHSP90-LRP1 and AKT-EphA2 signaling. An eHSP90/LRP1 signaling axis is required to sustain src directed AKT activation, AKT dependent P-EphA2_S897_, and LRP1 recruitment to EphA2. These signaling events facilitate lamellipodia formation and support GBM cell motility and invasion. Hypoxia amplifies eHsp90 signaling and corresponding motility via enhanced LRP1 expression and Hsp90 secretion. NPGA inhibits eHsp90 signaling, with consequent inhibition of AKT, disruption of EphA2 and LRP1 complexes, and blockade of cell motility. ephrin A1 ligand similarly suppresses AKT activation, P-EphA2_S897_, EphA2-LRP1 complexes, and elicits comparable inhibitory effects upon GBM cell motility and invasion.

Extracellular Hsp90 is emerging as a pivotal regulator of cell motility, invasion, and metastasis. Although the precise mechanisms of eHsp90 function remain largely unknown, eHsp90 regulates several well-established pro-motility molecules [19], [21], [23], [27]. Our current study linking eHsp90 signaling with EphA2-dependent cell motility and invasion adds a unique dimension to eHsp90's pro-tumorigenic repertoire. To our knowledge, this is the first report linking eHsp90-LRP1 signaling with EphA2 function. We show that eHsp90 promotes the recruitment of LRP1 to EphA2 in an AKT dependent manner and further demonstrate the previously unknown ability of LRP1 to exhibit specificity for a subset of AKT substrate proteins. Our data support a model whereby eHsp90-LRP1 dependent signaling is an obligate step for AKT activation and subsequent AKT directed phosphorylation of EphA2. This premise is supported by the shared ability of ephrin A1 ligand, NPGA or LRP1 silencing to suppress the phosphorylation of both AKT and EphA2, and to disrupt association between EphA2 and LRP1, culminating in the abrogation of lamellipodia formation and cell motility and invasion (Figure 6). Our studies therefore highlight a dual role for eHsp90 in transducing signaling via LRP1, while additionally promoting its LRP1 co-receptor functions to modulate EphA2 signaling, Recent reports highlighting the ability of eHsp90-LRP1 to elicit pro-motility function in normal and cancer cells [27], [42] portends a widespread role for this signaling pair in a variety of cancers that express EphA2. Whether eHsp90-LRP1 similarly regulates additional pro-motility receptors and intermediates is an area of active investigation.

The role of LRP1 in cell motility is controversial, with some reports documenting pro-motility function [24], [26], [27], [42], while others document anti-motility function [43]. These disparate reports may be ascribed to LRP1's complex role as both an endocytic and signaling receptor, coupled with its interaction with a diverse set of ligands [28]. Although LRP1 has been reported to confer pro-motility function in GBM cells [31] its mode of action is not well defined. Although we cannot exclude the possibility that eHsp90 influences LRP1 endocytic function, our data support a model whereby eHsp90-LRP1 functions as a signal transduction complex to regulate src dependent AKT phosphorylation, resulting in P-EphA2_S897_ directed cell motility and invasion. Interestingly, although a recent report demonstrated a requirement for eHsp90-LRP1 signaling in the motility and invasion of colon cancer cells, AKT did not participate in eHsp90 pro-motility function [27]. This example, in stark contrast to our model, highlights the signaling complexity inherent in cancer, and the ability of tumor cells to acquire dependence upon discrete components of eHsp90's signaling repertoire.

Our results implicate a role for eHsp90-LRP1 in serving as a central rheostat in controlling the amplitude of downstream signaling events. This was exemplified by the ability of eHsp90 protein to robustly induce the phosphorylation of src, AKT, and EphA2, concomitant with increased cell invasion. Importantly, we now show that eHsp90 signaling is amplified during cellular hypoxia, a well defined enhancer of cell motility and invasiveness in both normal and cancer cells [24], [40]. Hypoxia induces LRP1 gene expression in a number of cell types [44], although the physiological significance of this event has remained enigmatic. We show that hypoxia significantly increases the surface expression of both LRP1 and eHsp90, thereby amplifying the eHsp90-LRP1 signaling axis, evidenced by robust activation of src, AKT and EphA2, accompanied by enhanced cell motility and invasion. Strikingly, perturbation of eHsp90 function eliminates the hypoxia-mediated activation of these signaling intermediates and potently suppresses motility and invasion. Therefore, our data support the premise that the hypoxic potentiation of src/AKT/EphA2 activation is inextricably dependent upon eHsp90 directed cell motility and invasion.

Clinically, tissue hypoxia is a major contributor to several pathological features of GBM [45], [46], and our data implicate the hypoxic microenvironment of GBM as a significant potentiator of eHsp90-LRP1 signaling and GBM tumor cell aggressiveness. Given that AKT activation and expression of P-EphA2_S897_ are prevalent events in primary and recurrent GBM [10], [47], [48], and our data implicating LRP1 upregulation in clinical specimens, a model is proposed whereby GBM tumors amplify both eHsp90-LRP1 and AKT-EphA2 signaling axes to create a synergistic feed forward circuit that supports GBM aggressiveness. Our current findings significantly expand the known functions of eHsp90-LRP1 in malignancy and define crosstalk with AKT-EphA2 as a novel and essential mechanism for eHsp90-mediated pro-motility function in GBM. These unique insights into the effector molecules governing eHsp90 dependent invasive function in GBM highlight new approaches to curtail the aggressiveness associated with this malignancy.

## Materials and Methods

### Antibodies and Reagents

Antibodies to P-src_Y416_ (2101), src (2108), P-AKT_S473_ (4058), and AKT (9272) were purchased from Cell Signaling; goat and mouse EphA2 antibodies (AF3035, MAB3035) were from R&D Biosystems; Rabbit P-EphA2_S897_ anitbody was produced in Dr. Bingcheng Wang's laboratory [10] HA conjugated beads (11815016001) were from Roche; Protein G agarose beads (15920-010) were from Invitrogen, anti-phosphotyrosine antibody (PY20) was from Santa Cruz; mouse and rabbit Hsp90 antibodies (ADI-SPA-830, ADI-SPS-771) were from Assay Designs, and anti- alpha tubulin antibody (T6074) was from Sigma. The PE conjugated anti-Hsp90 (ADI-SPA-830PE) antibody was from Assay Designs and conjugated antibodies Alexa fluor 488 phalloidin (A12379) and 546 phalloidin (A22283) were from Invitrogen. Fluorescently conjugated secondary antibodies were purchased from Invitrogen (A-11001, A-11003, A-21050, A110055, A11008). Mouse monoclonal LRP1 antibody (11H4) was purified from a hybridoma cell line (CRL 1936) purchased from ATCC. The hybridoma supernatant was concentrated with a Vivacell 70 concentrator (Sartorius Biolab products) and purified with an NAb protein G antibody purification kit (Thermo Scientific) according to the manufacturer's instructions, and aliquots were stored at −20°C. Recombinant ephrin-A1-FC (602-A1-200) was purchased from R&D Biosystems. PP2 (529573) was from Calbiochem. Recombinant Hsp90 protein was obtained from Assay Designs (ADI-SPP-776). Geldanamycin was obtained from the Experimental Therapeutics Branch, National Cancer Institute, DMAG-N-oxide modified geldanamycin, (or non-permeable GA, NPGA) was synthesized by Zuping Xia (Pharmaceutical Sciences, Medical University of South Carolina).

### Cell Culture

The GBM cell lines U251 and U87 were obtained from ATCC, G48a and SV40 immortalized astrocytes were provided by Waldemar Debinski and Ashok Chauhan, respectively, and HA-AKT1-3 plasmids provided by Carola Neumann. The viral packaging cell line 293FT was from Invitrogen. Cells were maintained in their specified medium, supplemented with 10% fetal bovine serum, 1% HEPES and 1% penicilin/streptomycin in a 5% CO_2_-humidified atmosphere.

### Plasmids, siRNAs, and transfections

To construct HA-EphA2_S897G_, the following primers were used: GTGTCTATCCGGCTCCCCGGCACGAGCGGCTCGGAGG (upper), and CCTCCGAGCCGCTCGTGCCGGGGAGCCGGATAGACAC (lower). Primers were annealed to a wild type HA-EphA2 plasmid, and PCR was performed with *PfuUltra* HF DNA polymerase (Stratagene). Nonmutated parental DNA was cleaved with DpnI restriction enzyme and the reaction mix was used to transform XL1-Blue supercompetent bacteria. DNA was harvested from resultant clones and processed for sequence validation of the point mutation. Lentiviral particles against src, or nonspecific sequences, were purchased from Santa Cruz. To silence src, cells were infected with the viral particles (1∶200 dilution) in the presence of polybrene (8 µg/ml), and selection was performed with puromycin (Invivogen) for two weeks, whereupon surviving cells were pooled. To obtain shRNA viral particles for LRP1 and EphA2, 293FT cells were co-transfected with the viral packaging plasmids VSVG and PΔR 8.71, along with either shLRP1 or shEphA2. The cell medium was harvested at 48 hr the lentiviral supernatant was concentrated by ultracentrifugation, tittered, and 5×10^4^ particles were used to infect the recipient cells. Cells transduced with shEphA2 were selected in puromycin, while flow cytometry was used to isolate the highest expressing (95%) GFP-shLRP1 transduced cells. This selected population remained stable over time. All plasmid tranfections were performed with Lipofectamine 2000 (Invitrogen) according to the manufacturer's specifications.

### Cell motility and invasion assays

For cell wounding assays, a thin sterile pipette tip was used to create a scratch wound in confluent cell monolayers cultured in full serum. Pictures were taken at 0 and 24 hrs with an inverted Nikon eclipse TE 2000-S microscope with 10× magnification, and the extent of migration was calculated by measurement of the gap area using Image J software. For analysis of directional cell motility, chemotactic cell migration was carried out in modified Boyden chambers as previously described [9] with 2×10^4^ cells. The mean value from 5 fields per chamber was calculated from three independent experiments. Invasion assays were performed with 8-µm 24 well MatriGel-coated Transwell inserts (BD Biosciences) Inserts were rehydrated with medium for 2 hr at 37°C. Prior to plating, cells were serum starved for 16 hrs, and 4×10^5^ cells were subsequently plated in 0.1% serum containing medium. After incubation at 37°C for 16 hr cells were fixed and stained with 0.5% crystal violet. For all motility and invasion experiments, mitomycin C (5 ug/ml) (Sigma) was added at the time of plating to suppress proliferation. Cells migrating through both the Matrigel and the filter pores were counted from 5 random fields from 3 wells and represented as a mean (± SD) of three replicates. P value less than 0.05 was considered significant.

### Flow Cytometery

Cells were trypsinized, washed with PBS, blocked (0.1% sodium azide, 2% bovine serum albumin/PBS), and Hsp90 was detected with PE conjugated antibody, EphA2 was detected with anti-goat antibody and LRP1 was detected with the mouse 11H4 antibody, all diluted 1∶50 in blocking buffer. Washed cells were then incubated with the appropriate labeled secondary antibody and resuspended in PBS. Data were acquired with a FACS Calibur4-color flow cytometer (BD, Biosciences), and analyzed with FlowJo software (TreeStar). A minimum of 10,000 cells was counted per experiment. Negative controls consisted of cells incubated either in the complete absence of antibody or with isotype matched secondary antibody alone. The mean fluorescence intensity (MFI) of the signal was calculated by Flow Jo software and signal obtained from EphA2 and LRP1 was normalized with that obtained from isotype controls.

### Western blot and Immunoprecipitation

Cell extracts were prepared as described [11]. For immunoprecipitation experiments, cells were lysed (10 mM Tris pH7.4, 150 mM NaCl, 0.5% NP40, 10% glycerol, 2 mM EDTA with protease inhibitor cocktail (Roche), immunoprecipitates eluted with 6× SDS loading dye, and densitometric analyses performed with ImageJ software.

### Hypoxic treatment

For hypoxia treatments, cells were placed within an enclosed bactron anaerobic chamber (Shel Lab) containing a 37°C temperature controlled incubator. The incubator was humidified with a water tray and the oxygen concentration was maintained via regulated infusion of premixed gas (94% N_2_, 5% CO_2_, and 1% O_2_). The oxygen concentration within the chamber was continuously monitored.

### Hsp90α ELISA

To detect expression of secreted Hsp90α, equivalent cell numbers (2×10^5^) were plated overnight and replenished with complete media 24 hr prior to harvest. Conditioned medium was collected, debris removed by centrifugation (5 min, 1200 x g) and Hsp90 levels detected with an Hsp90α ELISA kit (Assay Designs). Background values (from control medium) were subtracted from readings (examined in triplicate) and values are presented as the average ng of Hsp90 per ml of conditioned medium with the standard deviation shown.

### Immunohistochemistry

Banked tissues were acquired through the Hollings Cancer Center Tissue Biorepository (Medical University of South Carolina), or the Wake Forest Brain Tumor Center of Excellence (BTCOE). Commercial GBM TMAs (T171, GL805, GL2083) were obtained from US Biomax, Inc. Embedded tissues were deparaffinized and antigen retrieval was performed with Target Retrieval Solution (DAKO, Carpinteria, CA) coupled with steaming. Banked frozen GBM tissue was serially sectioned (5 µm), fixed in acetone, hydrated in PBS and subjected to immunostaining. The slides were incubated with the indicated primary antibodies: goat polyclonal EphA2 (1∶500), P-EphA2_S897_ (1∶500), LRP1 (11H4, 1∶500–1∶1000) and signal visualized with a biotinylated secondary antibody (1∶200) and streptavidin biotin peroxidase kit (DAKO LSAB+ System-HRP), along with a DAB chromagen and peroxide substrate. For BTCOE images, immunostaining was visualized using an ABC *Elite* Kit (Vector Labs) followed by DAB without nickel (black, EphA2), DAB (brown, LRP1) or VectorRed (P-EphA2_S897_) as the chromagen (Vector Labs). For negative control immunostaining, nonspecific, species matched biotinylated antibodies were added in tandem in the absence of primary antibody. All final images were independently verified by a pathologist as representing similar fields from adjacent tissue sections. Images were acquired at 200× magnification with an Eclipse 55i Nikon Digital photomicroscope system, or at 400× (BTCOE series) under oil immersion. For histological assessment, pathological scoring was performed - negative staining (0), weak staining (1), moderate (2–3), and strong (4–5). Fisher's exact test was used to analyse association between sample type and LRP1 expression level. P value less than 0.05 was considered as a significant.

### Immunofluorescence

To image lamellipodia formation, confluent monolayers were scratch wounded, and 4 hr later, cells were fixed with 4% paraformaldehyde and permeabilized with 0.1% Triton X-100 in PBS. Immunofluorescence was performed as described in [11].

### Statistical analysis

All cell motility and invasion experiments were performed in triplicate. Data shown are presented as means ± SD; differences in treatment groups are defined as statistically significant at P<0.05 value, as calculated from Student's t test.

## Supporting Information

Figure S1 **Interference with eHsp90 signaling inhibits GBM cell motility and invasion.** (A) Relative degree of LRP1 suppression in stably selected LRP1 silenced G48a cells. LRP1 was immunodetected from equivalent amounts of lysate. (B) GBM cell motility is inhibited by either LRP1 silencing or NPGA treatment (16 hr). Representative images from Boyden cell motility experiments. Serum within the lower wells served as the chemoattractant. (C) GBM cell invasion is suppressed by either LRP1 silencing or NPGA treatment. Representative images from Matrigel invasion assays, performed with conditions as above. (D) Surface expression of Hsp90 and LRP1 is elevated in GBM as compared to normal astrocytes. eHsp90 and LRP1 were detected in the indicated GBM cell lines (G48a, U87, U251) or immortalized astrocytes (SVGA) by flow cytometric analysis of nonpermeabilized cells. Surface Hsp90 was visualized with PE conjugated Hsp90 antibody, relative to matched isotype control, and LRP1 detection was facilitated with anti-LRP1 antibody, followed by fluorescently labeled secondary antibody. Positively stained cells are represented as the area under the respective histogram, and mean fluorescence intensity (MFI) values are shown. (E) Hsp90α is secreted from GBM cell lines. An ELISA assay was utilized to detect the levels of Hsp90α in conditioned medium from equivalent cell numbers (1×10^6^). (F) Relative cellular expression of LRP1, Hsp90α, and EphA2 in SVGA and GBM cell lines. Cell extracts were harvested from the indicated panel of cell lines and tubulin was used as a protein loading control. (G) Surface Hsp90 expression is diminished by either LRP1 silencing or NPGA treatment. Flow cytometric analysis was performed as in D, except that, where indicated, cells were treated with NPGA for 16 hr prior to analysis. Surface Hsp90 expression was relatively proportional to surface LRP1 expression, as demonstrated by LRP1 silencing. Although NPGA reduced surface Hsp90 expression, surface LRP1 expression was not affected.
(TIF)

Figure S2 **eHsp90-LRP1 regulates EphA2 dependent motility, invasion and signaling.** (A) Representative immunoblot showing the extent of EphA2 suppression following stable transduction of shEphA2 in G48a cells. (B) Analysis of the effects of EphA2 silencing upon G48a cell motility in the presence or absence of NPGA. Confluent monolayers of parental or EphA2 silenced cells were scratched and representative images of wounded areas are shown from time 0 and 16 hr post wounding. The graph is represented as the mean (± SD) of three replicates. *p<0.001. (C, D) The anti-motility and anti-invasive effects of NPGA upon parental and EphA2 silenced cells were evaluated with Boyden chamber (C) or Matrigel (D) assays. Experiments were performed as in Figures 1C and 1D, and representative images shown. (E) Interference with eHsp90 signaling by NPGA or LRP1 silencing suppressed src phosphorylation. (F) Representative degree of src suppression following stable transduction of src shRNA lentiviral construct in G48a cells. (G) Antibody-mediated Hsp90 targeting suppresses P-src_Y418_, P-AKT_S473_ and P-EphA2_S897_. G48a cells were incubated for 16 hr with either control antibody (IgG), or anti-Hsp90α antibody (SPS-771, 20 ug/ml) followed by immunoblot analysis for the indicated proteins. Where indicated, ephrin A1 was added 10 min prior to cell lysis. (H) Interference with eHsp90 signaling does not alter surface EphA2 expression. Flow cytometry was performed on intact G48a cells to compare EphA2 surface expression in parental G48a cells, relative to LRP1 silenced or NPGA treated cells (16 hr). EphA2 protein was detected by a rabbit polyclonal antibody recognizing an extracellular epitope, followed by fluorescently labeled anti-goat antibody. Representative histograms of EphA2 staining are shown. A fluorescently labeled isotype matched control antibody was included to demonstrate EphA2 signal specificity.
(TIF)

Figure S3 **Preservation of AKT activation is required for lamellipodia formation, and concomitant cell motility and invasion.** (A) A scratch wound assay was utilized to evaluate the ability of either native or Hsp90_ΔATP_ to rescue G48a cell motility in the presence of NPGA. Cells were treated with either native or Hsp90_ΔATP_ proteins (3 µg/ml) for 16 hr and representative images (10× magnification) are shown. The graph is represented as the mean (± SD) of three replicates. *p<0.001. (B) Native or Hsp90_ΔATP_ proteins (3 µg/ml) were added (top and bottom wells) to serum starved G48a cells in a Matrigel invasion assay. Representative images are shown. (C) The indicated Hsp90 proteins were added (15 min) to serum starved G48a cells 4 hr post cell wounding, as in Figure 2C. Cells were continuously exposed to NPGA 16 hr prior to fixation. Expression of P-EphA2_S897_, F-actin, and the co-localization of these proteins were analyzed by confocal microscopy. Scale bar is 25 µm. (D) A scratch wound assay was utilized to evaluate the ability of constitutively active (myristolyated) AKT isoforms to sustain G48a cell motility in the presence of NPGA. Cells were treated as in Figure S2B and representative images shown.
(TIF)

Figure S4 **Hypoxia stimulates GBM motility and invasion via eHsp90 dependent signaling.** (A) The effects of hypoxia upon G48a cell motility was evaluated in either the presence or absence of NPGA by scratch wound assay, Data is represented as the mean (± SD) of three replicates. *p<0.001. (B) Cell invasion was determined by a Matrigel assay following exposure of G48a cells to normoxia or hypoxia (1% O_2_) for 16 hr in the presence or absence of NPGA. Representative images are shown.
(TIF)

Figure S5 **Point mutated HA-EphA2_S897G_ is not recognized by the P-EphA2_S897_ specific antibody.** U87 cells were transiently transfected with the indicated HA-tagged EphA2 plasmids, and EphA2 activation status was evaluated by probing HA immunopurified extracts with P-EphA2_S897_ antibody. Expression levels of transduced proteins were verified by probing total cellular lysate with HA antibody.
(TIF)
