# Supplementary figures and images for: A Novel Extracellular Hsp90 Mediated Co-Receptor Function for LRP1 Regulates EphA2 Dependent Glioblastoma Cell Invasion
Source: PLoS One. 2011 Mar 8;6(3):e17649. doi: 10.1371/journal.pone.0017649 (PMC3050925; doi:10.1371/journal.pone.0017649)

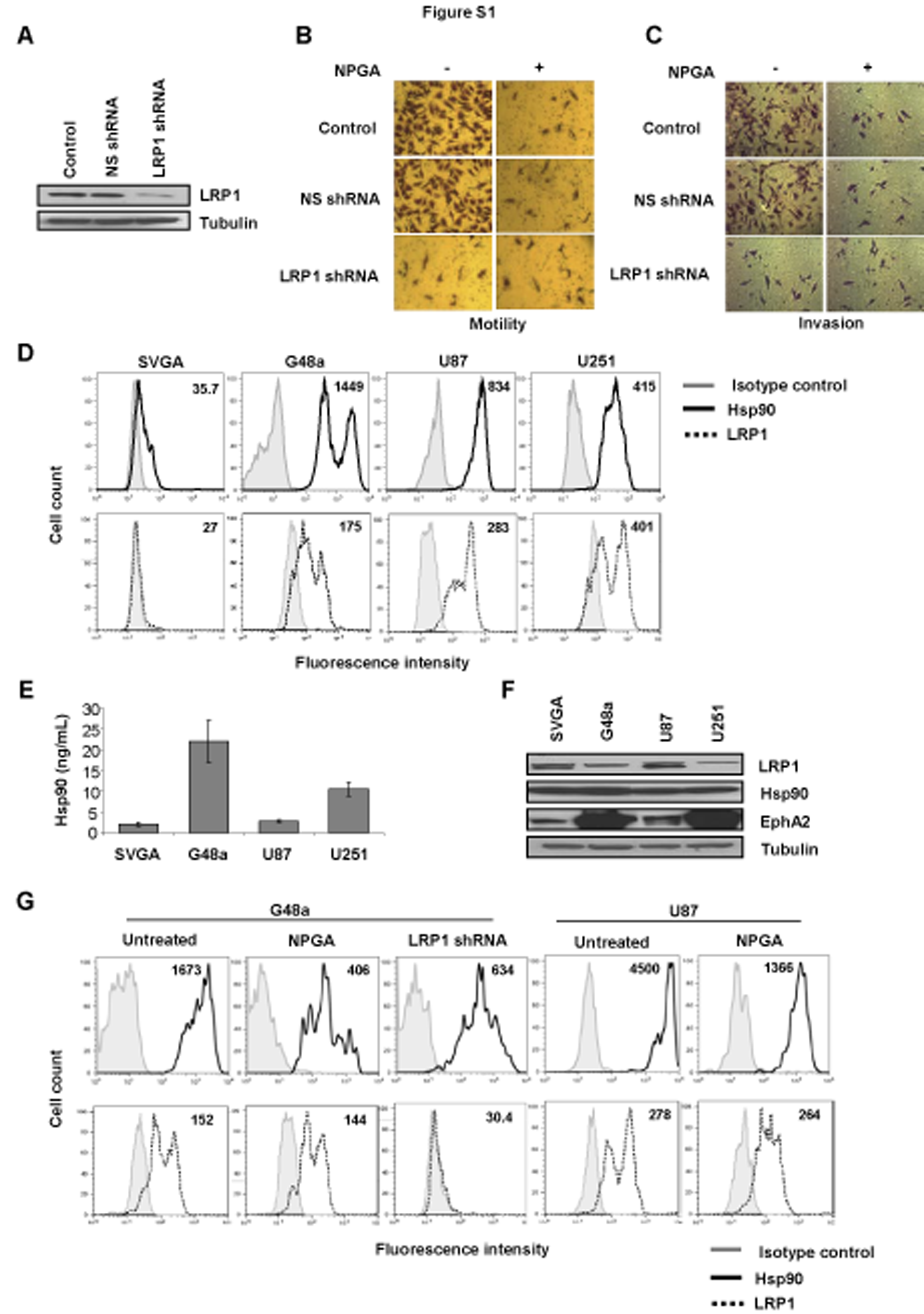

Supplement: Figure S1 — Interference with eHsp90 signaling inhibits GBM cell motility and invasion. (A) Relative degree of LRP1 suppression in stably selected LRP1 silenced G48a cells. LRP1 was immunodetected from equivalent amounts of lysate. (B) GBM cell motility is inhibited by either LRP1 silencing or NPGA treatment (16 hr). Representative images from Boyden cell motility experiments. Serum within the lower wells served as the chemoattractant. (C) GBM cell invasion is suppressed by either LRP1 silencing or NPGA treatment. Representative images from Matrigel invasion assays, performed with conditions as above. (D) Surface expression of Hsp90 and LRP1 is elevated in GBM as compared to normal astrocytes. eHsp90 and LRP1 were detected in the indicated GBM cell lines (G48a, U87, U251) or immortalized astrocytes (SVGA) by flow cytometric analysis of nonpermeabilized cells. Surface Hsp90 was visualized with PE conjugated Hsp90 antibody, relative to matched isotype control, and LRP1 detection was facilitated with anti-LRP1 antibody, followed by fluorescently labeled secondary antibody. Positively stained cells are represented as the area under the respective histogram, and mean fluorescence intensity (MFI) values are shown. (E) Hsp90α is secreted from GBM cell lines. An ELISA assay was utilized to detect the levels of Hsp90α in conditioned medium from equivalent cell numbers (1×10^6^). (F) Relative cellular expression of LRP1, Hsp90α, and EphA2 in SVGA and GBM cell lines. Cell extracts were harvested from the indicated panel of cell lines and tubulin was used as a protein loading control. (G) Surface Hsp90 expression is diminished by either LRP1 silencing or NPGA treatment. Flow cytometric analysis was performed as in D, except that, where indicated, cells were treated with NPGA for 16 hr prior to analysis. Surface Hsp90 expression was relatively proportional to surface LRP1 expression, as demonstrated by LRP1 silencing. Although NPGA reduced surface Hsp90 expression, surface LRP1 expression was [file pone.0017649.s001.tif]

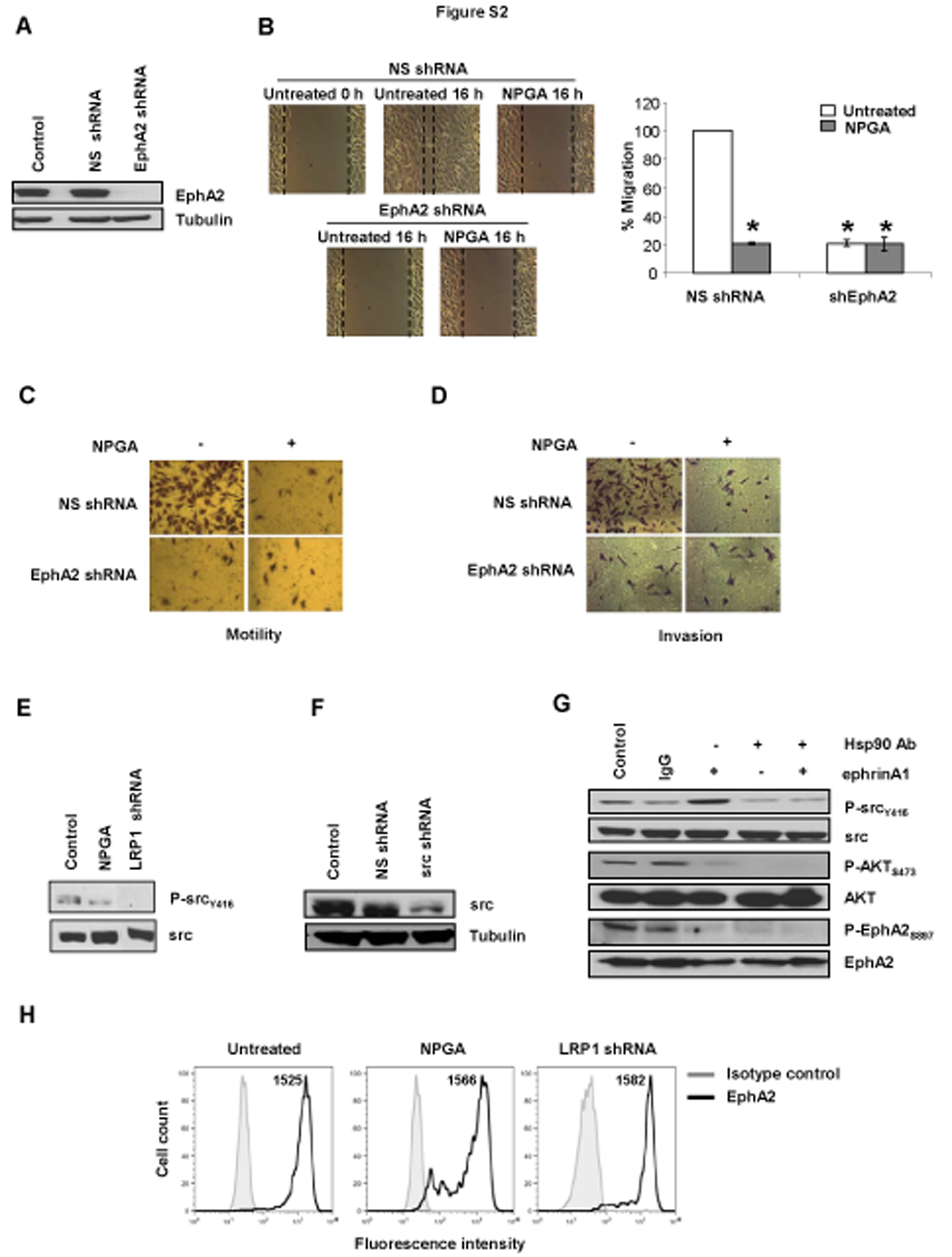

Supplement: Figure S2 — eHsp90-LRP1 regulates EphA2 dependent motility, invasion and signaling. (A) Representative immunoblot showing the extent of EphA2 suppression following stable transduction of shEphA2 in G48a cells. (B) Analysis of the effects of EphA2 silencing upon G48a cell motility in the presence or absence of NPGA. Confluent monolayers of parental or EphA2 silenced cells were scratched and representative images of wounded areas are shown from time 0 and 16 hr post wounding. The graph is represented as the mean (± SD) of three replicates. *p<0.001. (C, D) The anti-motility and anti-invasive effects of NPGA upon parental and EphA2 silenced cells were evaluated with Boyden chamber (C) or Matrigel (D) assays. Experiments were performed as in Figures 1C and 1D, and representative images shown. (E) Interference with eHsp90 signaling by NPGA or LRP1 silencing suppressed src phosphorylation. (F) Representative degree of src suppression following stable transduction of src shRNA lentiviral construct in G48a cells. (G) Antibody-mediated Hsp90 targeting suppresses P-srcY418, P-AKTS473 and P-EphA2S897. G48a cells were incubated for 16 hr with either control antibody (IgG), or anti-Hsp90α antibody (SPS-771, 20 ug/ml) followed by immunoblot analysis for the indicated proteins. Where indicated, ephrin A1 was added 10 min prior to cell lysis. (H) Interference with eHsp90 signaling does not alter surface EphA2 expression. Flow cytometry was performed on intact G48a cells to compare EphA2 surface expression in parental G48a cells, relative to LRP1 silenced or NPGA treated cells (16 hr). EphA2 protein was detected by a rabbit polyclonal antibody recognizing an extracellular epitope, followed by fluorescently labeled anti-goat antibody. Representative histograms of EphA2 staining are shown. A fluorescently labeled isotype matched control antibody was included to demonstrate EphA2 signal specificity. (TIF) [file pone.0017649.s002.tif]

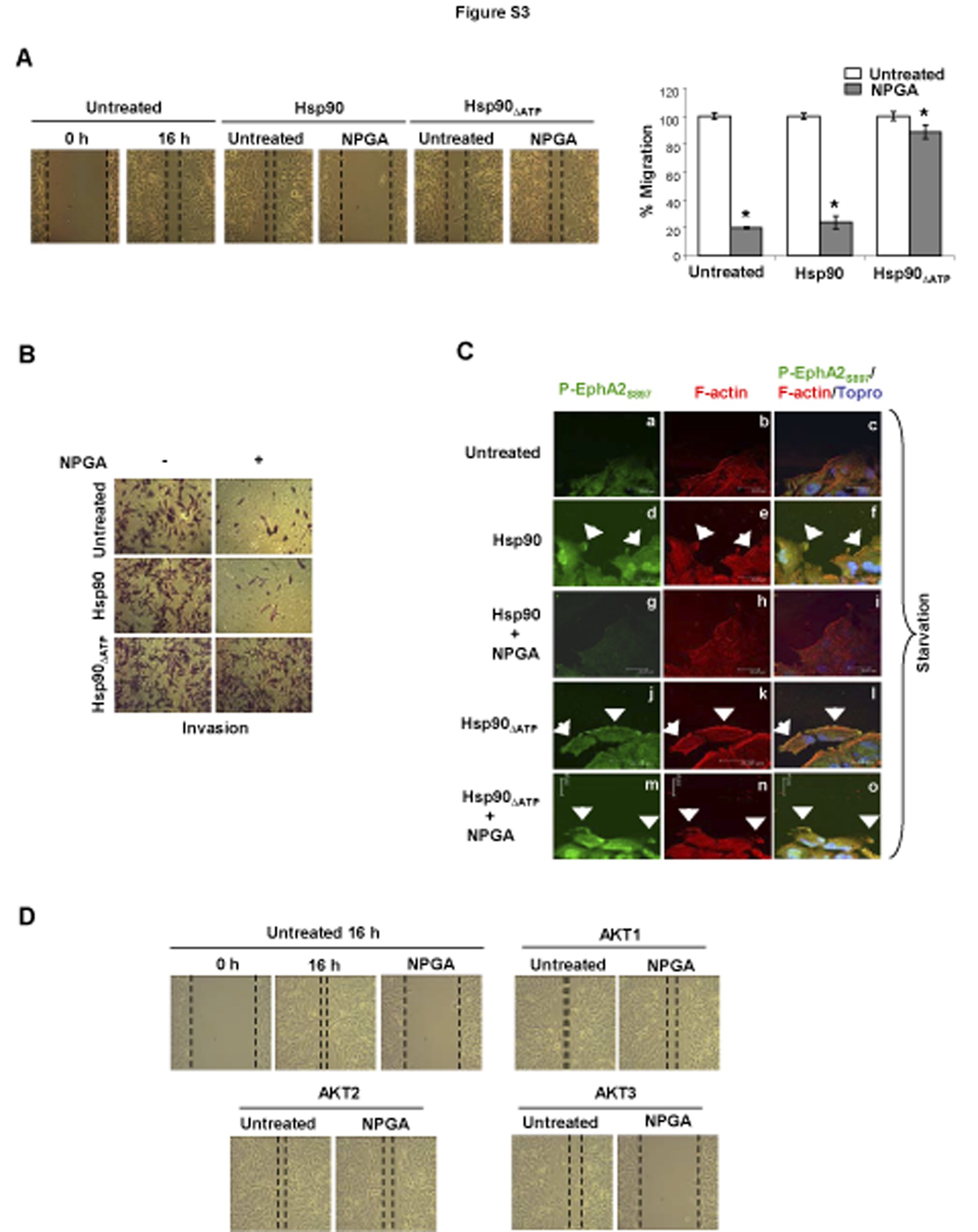

Supplement: Figure S3 — Preservation of AKT activation is required for lamellipodia formation, and concomitant cell motility and invasion. (A) A scratch wound assay was utilized to evaluate the ability of either native or Hsp90ΔATP to rescue G48a cell motility in the presence of NPGA. Cells were treated with either native or Hsp90ΔATP proteins (3 µg/ml) for 16 hr and representative images (10× magnification) are shown. The graph is represented as the mean (± SD) of three replicates. *p<0.001. (B) Native or Hsp90ΔATP proteins (3 µg/ml) were added (top and bottom wells) to serum starved G48a cells in a Matrigel invasion assay. Representative images are shown. (C) The indicated Hsp90 proteins were added (15 min) to serum starved G48a cells 4 hr post cell wounding, as in Figure 2C. Cells were continuously exposed to NPGA 16 hr prior to fixation. Expression of P-EphA2S897, F-actin, and the co-localization of these proteins were analyzed by confocal microscopy. Scale bar is 25 µm. (D) A scratch wound assay was utilized to evaluate the ability of constitutively active (myristolyated) AKT isoforms to sustain G48a cell motility in the presence of NPGA. Cells were treated as in Figure S2B and representative images shown. (TIF) [file pone.0017649.s003.tif]

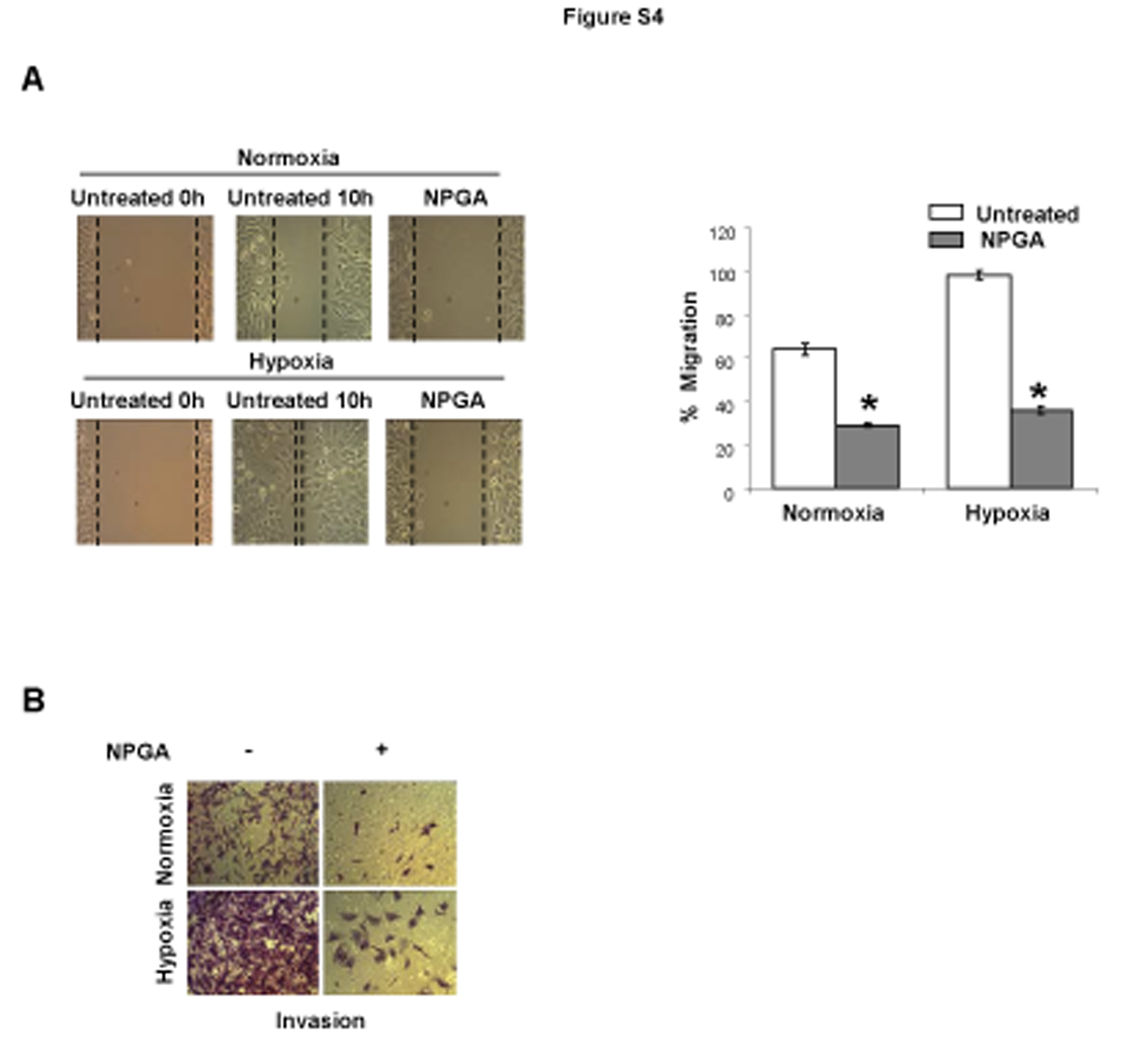

Supplement: Figure S4 — Hypoxia stimulates GBM motility and invasion via eHsp90 dependent signaling. (A) The effects of hypoxia upon G48a cell motility was evaluated in either the presence or absence of NPGA by scratch wound assay, Data is represented as the mean (± SD) of three replicates. *p<0.001. (B) Cell invasion was determined by a Matrigel assay following exposure of G48a cells to normoxia or hypoxia (1% O2) for 16 hr in the presence or absence of NPGA. Representative images are shown. (TIF) [file pone.0017649.s004.tif]

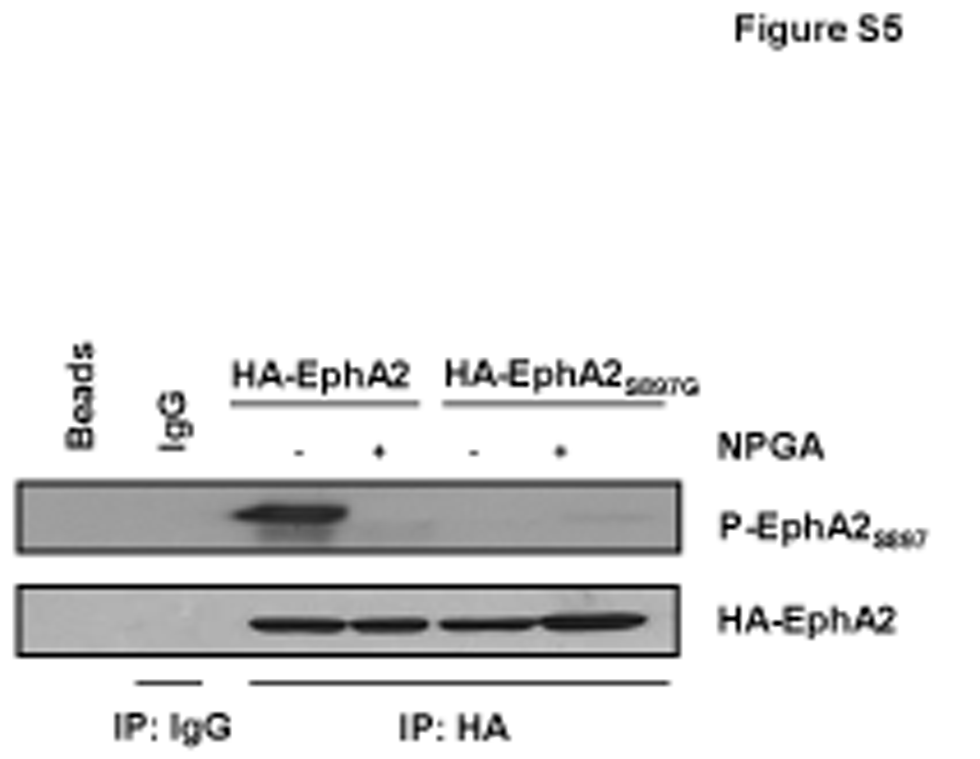

Supplement: Figure S5 — Point mutated HA-EphA2S897G is not recognized by the P-EphA2S897 specific antibody. U87 cells were transiently transfected with the indicated HA-tagged EphA2 plasmids, and EphA2 activation status was evaluated by probing HA immunopurified extracts with P-EphA2S897 antibody. Expression levels of transduced proteins were verified by probing total cellular lysate with HA antibody. (TIF) [file pone.0017649.s005.tif]
